# Quantification of Lysogeny Caused by Phage Coinfections in Microbial Communities from Biophysical Principles

**DOI:** 10.1128/mSystems.00353-20

**Published:** 2020-09-15

**Authors:** Antoni Luque, Cynthia B. Silveira

**Affiliations:** a Department of Mathematics and Statistics, San Diego State University, San Diego, California, USA; b Viral Information Institute, San Diego State University, San Diego, California, USA; c Computational Science Research Center, San Diego State University, San Diego, California, USA; d Department of Biology, University of Miami, Coral Gables, Florida, USA; Vanderbilt University

**Keywords:** stochastic biophysical model, lysogeny, microbial abundance, multiplicity of infection, adsorption rates, commitment time

## Abstract

Temperate phages can associate with their bacterial host to form a lysogen, often modifying the phenotype of the host. Lysogens are dominant in the microbially dense environment of the mammalian gut. This observation contrasts with the long-standing hypothesis of lysogeny being favored at low microbial densities, such as in oligotrophic marine environments. Here, we hypothesized that phage coinfections—a well-understood molecular mechanism of lysogenization—increase at high microbial abundances. To test this hypothesis, we developed a biophysical model of coinfection for marine and gut microbiomes. The model stochastically sampled ranges of phage and bacterial concentrations, adsorption rates, lysogenic commitment times, and community diversity from each environment. In 90% of the sampled marine communities, less than 10% of the bacteria were predicted to be lysogenized via coinfection. In contrast, 25% of the sampled gut communities displayed more than 25% of lysogenization. The probability of lysogenization in the gut was a consequence of the higher densities and higher adsorption rates. These results suggest that, on average, coinfections can form two trillion lysogens in the human gut every day. In marine microbiomes, which were characterized by lower densities and phage adsorption rates, lysogeny via coinfection was still possible for communities with long lysogenic commitment times. Our study indicates that different physical factors causing coinfections can reconcile the traditional view of lysogeny at poor host growth (long commitment times) and the recent Piggyback-the-Winner framework proposing that lysogeny is favored in rich environments (high densities and adsorption rates).

**IMPORTANCE** The association of temperate phages and bacterial hosts during lysogeny manipulates microbial dynamics from the oceans to the human gut. Lysogeny is well studied in laboratory models, but its environmental drivers remain unclear. Here, we quantified the probability of lysogenization caused by phage coinfections, a well-known trigger of lysogeny, in marine and gut microbial environments. Coinfections were quantified by developing a biophysical model that incorporated the traits of viral and bacterial communities. Lysogenization via coinfection was more frequent in highly productive environments like the gut, due to higher microbial densities and higher phage adsorption rates. At low cell densities, lysogenization occurred in bacteria with long duplication times. These results bridge the molecular understanding of lysogeny with the ecology of complex microbial communities.

## Abstract (precis)

The association of temperate phages and bacterial hosts during lysogeny manipulates microbial dynamics from the oceans to the human gut. Lysogeny is well studied in laboratory models, but its environmental drivers remain unclear. Here, we quantified the probability of lysogenization caused by phage coinfections, a well-known trigger of lysogeny, in marine and gut microbial environments. Coinfections were quantified by developing a biophysical model that incorporated the traits of viral and bacterial communities. Lysogenization via coinfection was more frequent in highly productive environments like the gut, due to higher microbial densities and higher phage adsorption rates. At low cell densities, lysogenization occurred in bacteria with long duplication times. These results bridge the molecular understanding of lysogeny with the ecology of complex microbial communities.

## INTRODUCTION

Temperate phages can integrate into their host’s genome as a prophage or persist as extrachromosomal elements forming a lysogen. Half of the genomes that have been sequenced from bacterial isolates contain prophages (1–3). Most lysogens display changes in phenotypes, such as protection against other phage infections and additional metabolic functions (4–6). Despite lysogeny’s profound impacts on the structure and functioning of microbial communities, its environmental drivers remain unclear.

Currently, the best proxies to estimate the frequency of lysogeny in microbial communities are the distributions of integrases, excisionases, lysis repressors, and sequences with high similarity with reference prophages (7, 8). The abundance of these markers in metagenomic data indicates that microbially dense environments, such as the mammalian gut, are dominated by temperate phages and bacterial lysogens (9–14). The high frequency of lysogeny in the gut can be explained by the Piggyback-the-Winner (PtW) framework, which proposes that the phenotypic advantages of lysogeny are favored at high host abundances (15–17).

Compared to animal-associated microbial communities, aquatic marine ecosystems have much weaker lysogenic signatures (7, 15). Genomic analyses have indicated that lysogeny is frequent in deep oligotrophic waters (18–20). In these environments where bacterial abundances are low, ranging from 10^4^ to 10^5^ cells per ml, 98% of the temperate viral sequences observed in the cellular metagenomes are also found in the virome (7). The increase in lysogeny in marine ecosystems with low productivity has been historically hypothesized to serve as a low-density refugium for phages during poor host growth (21). However, highly productive marine ecosystems where abundances of bacteria rise above 10^6^ cells per ml also increase their lysogenic signatures, following the PtW framework (15). This is consistent with the observation that high intrinsic growth rates are the most important predictor of the frequency of prophages in bacteria with complete genomes sequenced (1, 22). Growth rates alone cannot explain the prevalence of lysogeny under both high- and low-productivity conditions, suggesting that density-dependent factors might play a role.

Phage coinfections promote lysogenization in model phage and bacteria under both poor and rich growth conditions (23–25). In lambda phage, the percentage of lysogenized cells increases from 0.1% to close to 100% when the multiplicity of infection (MOI) increases from 0.05 to 100 phages per bacteria (26–30). Coinfections increase the expression of the lambda repressors of the lytic pathway and activate a cascade of genes responsible for phage integration (31–33). The response of lysogeny to coinfection seems to represent a widespread strategy among temperate phage populations. Most temperate phages encode a repressor system functionally similar to lambda’s cro/cI (34). For example, phage P22, which displays only 13% genomic similarity with lambda, encodes the same repressor system (35). Even phages such as Mu and Epsilon15, with completely distinct molecular mechanisms for the control of lysogeny, also increase lysogeny at higher rates of coinfection (36). Yet, the extent to which coinfections occur in complex microbial communities, and their impact on lysogeny, has not been quantified.

After its first introduction, the term MOI became known in the field as the initial ratio of phage particles (P_0_) to bacterial cells (B_0_) added to an experiment (MOI = P_0_/B_0_). This definition of MOI, however, does not necessarily capture the effective number of coinfections, which also depends on the chances of encounter between the phage and the host (37). A recent stochastic model shows that at the single-cell level, the average number of coinfections is primarily determined by the phage concentrations and phage decision times (38). Because highly productive environments have higher phage concentrations, here we hypothesize that the prevalence of lysogeny in these environments is a consequence of an increase in coinfections.

To test this hypothesis, a biophysical model was derived to incorporate the physical traits that determine phage (co)infection (COI) and its associated probability of lysogeny. The model was simulated for a range of phage and bacterial abundances, community diversity, adsorption rates, and lysogenic commitment times from marine and mammalian gut communities. The availability of large amounts of public data from these two ecosystems allowed us to test the hypothesis across a wide range of microbial densities.

## RESULTS

### Relationship between COI and phage-to-bacterium ratios.

**FIG 1:**
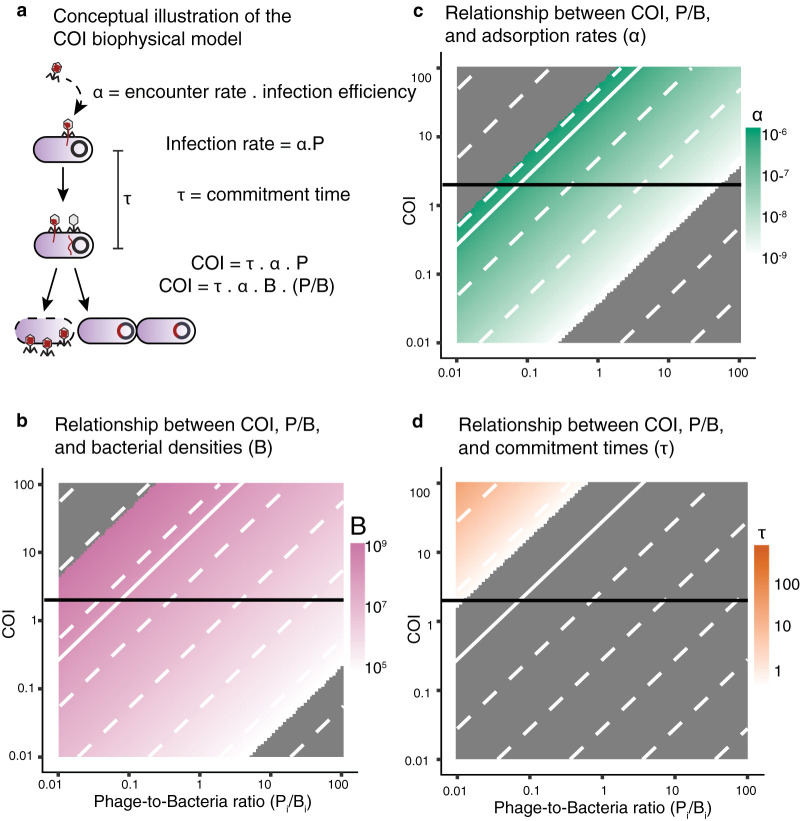
Relationship between number of (co)infections and phage-to-bacterium ratios. (a) Illustration of the derivation and parameter description for the biophysical (co)infection (COI) model, equation 1. COI was defined as the average number of phages infecting a cell within the commitment time (τ). COI = 1 means one phage infection. COI = 2 means two phage infections. (b to d) Relationship between COI and phage-to-bacterium ratios as a function of bacterial abundances (b), adsorption rates (c), and lysogenic commitment times (d). Panels b to d are contour plots of the quantitative outputs of the dependencies from equation 1. The color gradients cover the environmental ranges of values for each of these parameters: bacterial concentrations (pink), adsorption rates (green), and lysogenic commitment times (orange). (b to d) The dashed white lines indicate constant values of the parameters in the gradient scale. The gray areas correspond to values beyond the environmental ranges of bacterial concentrations, adsorption rates, and commitment times obtained from the meta-analysis of marine and gut ecosystems. The horizontal black line indicates COI = 2, that is, two average phage infections within the commitment time (τ). The solid white line indicates the median values for the bacterial concentration (B_0_), adsorption rates (α_0_), and lysogenic commitment time (τ_0_) for lambda in laboratory experiments.

The model introduced in equation 1 was reexpressed to estimate the average number of phage (co)infections (COI) in terms of the phage-to-bacterium ratios (P*_i_*/B*_i_*) for a single phage-host pair (Fig. 1a). The phage-to-bacterium ratio (P*_i_*/B*_i_*) was used as a proxy for the operational multiplicity of infection (MOI = P_0_/B_0_) widely used in the phage field. The bacterial densities, adsorption rates, and commitment times were plotted as a function of COI and P*_i_*/B*_i_* (Fig. 1b to d). To illustrate the relationship of COI with different physical parameters, only one value was varied at a time covering typical environmental ranges (see meta-analysis section for environmental ranges). COI was higher for higher bacterial concentrations (Fig. 1b), phage adsorption rate constants (Fig. 1c), and lysogenic commitment times (Fig. 1d). For the typical adsorption rate and commitment time of lambda, the model showed that an average of two or more phage infections (COI ⩾ 2) was unlikely to occur at bacterial densities below 10^6^ cells/ml, even for phage-to-bacterium ratios above 10 (bottom right values in Fig. 1b). Due to the high adsorption rate of lambda, the average number of coinfections generated per phage-to-bacterium ratio was near the upper range of environmental values (Fig. 1c). Due to the short lysogenic commitment time of lambda, the average coinfections per phage-to-bacterium ratio unit were below the environmental values (Fig. 1d). These results show that as one departs from ideal experimental conditions, the proxy for MOI did not capture the average number of coinfections.

**FIG 2:**
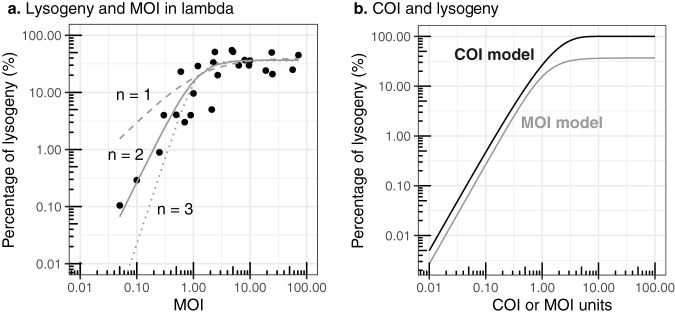
Comparison of percentage of lysogeny for lambda MOI and COI model. (a) Relationship between the percentage of lysogeny and MOI (initial phage-to-bacterium ratio) in lambda-E. coli experiments (28, 29). The lines correspond to fitted Hill-Langmuir cooperation models of order *n* =1 (dashed), *n* = 2 (solid), and *n* = 3 (dotted). (b) Percentage of lysogeny estimated from the coinfection probability model as a function of COI, equation 2 (solid black line). Hill-Langmuir model of order *n* = 2 from panel a as a function of MOI (solid gray line).

The probability of lysogeny as a function of average coinfections (COI) was compared with lambda-Escherichia coli MOI experiments. The percentage of lysogenized cells increased as a function of MOI and was best described by a sigmoidal Hill-Langmuir equation of order *n* = 2, compared to orders *n* = 1 and *n* = 3 (Fig. 2a). This equation implied that two phages cooperated in producing lysogeny, in agreement with single-molecule experiments (25). This empirical model was functionally similar to the predicted probability of lysogeny from the average coinfection Poisson model, equation 2, which assumed that at least two infections were necessary to produce lysogeny (Fig. 2b). The MOI and COI values were similar. The discrepancy between the maximum percentages of lysogeny for the MOI and COI models was due to the fact that the COI model was a function of the initial phage-to-bacterium ratio and did not account for the rapid removal of phage particles due to cell adsorption in the course of the experiments. The MOI-COI equivalence in lambda-*E. coli* experiments was due to the fact that the original MOI experiments were set up to capture the number of coinfections, which was only possible for those specific growth conditions (Fig. 1).

### Meta-analysis of COI physical parameters from marine and animal ecosystems.

**FIG 3:**
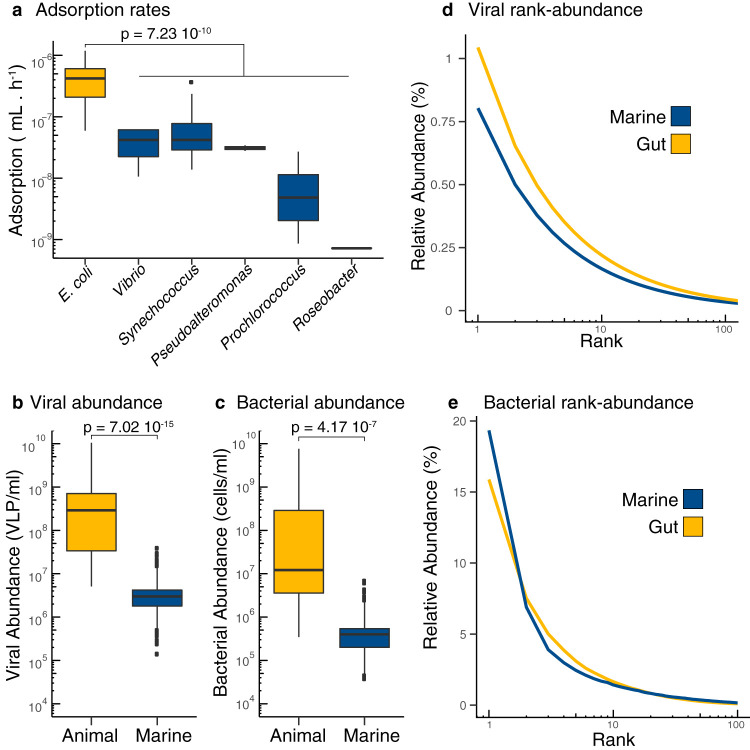
Meta-analysis of COI parameters in marine and animal microbiomes. (a) Adsorption rates from single phage-host pairs of bacterial hosts derived from the mammalian gut (yellow) and marine environments (blue). The same color coding applies to all panels. (b and c) Viral abundances (b) and bacterial abundances (c) per ml in animal-associated and marine environments. (d) Rank-abundance curve displaying the top 100 ranks of phage genotypes identified in metagenomic data from human gut and marine samples. (e) Rank-abundance curve displaying the top 100 ranks of bacterial genotypes identified in metagenomic data from human gut and marine samples. The references for each original study, and the values for each data point are provided as Data Sets S1 to S5.

To apply the biophysical COI model to microbial communities and estimate lysogeny generated by phage coinfections, the ranges of phage adsorption rate constants, lysogenic commitment times, and phage-bacterium pair abundances were determined for marine and animal ecosystems. The range of adsorption rates was 7.2 · 10^−10^ to 3.7 · 10^−7^ ml/h for marine phages infecting *Prochlorococcus* sp., *Roseobacter* sp., *Pseudoalteromonas* sp., *Synechococcus* sp., and *Vibrio* sp. (Fig. 3a). The range of adsorption rates was 5.9 · 10^−8^ to 1.2 · 10^−6^ ml/h for gut phages infecting E. coli. The median adsorption rate for gut phages was 1 order of magnitude higher (4.2 · 10^−7^ ml/h) than the median for marine phages (3.4 · 10^−8^ ml/h [Fig. 3a], and *t* test *P* = 7.23 · 10^−10^). Lysogenic commitment times were longer in marine communities, 11 to 808 h, than in the mammalian gut, 2.74 to 7.27 h. This was a consequence of the long duplication times of marine communities in their natural environment. The phage and bacterium pair abundances were determined by combining the total and relative abundances of phage and bacteria in each ecosystem. Phage abundances were 1.4 · 10^5^ to 3.7 · 10^7^ phages/ml (marine) and 5.1 · 10^6^ to 1.1 · 10^10^ phages/ml (animal) (Fig. 3b). Bacterial abundances ranged from 3.8 · 10^4^ to 6.8 · 10^6^ cells/ml (marine) and from 3.5 · 10^5^ to 7.7 · 10^9^ cells/ml (animal) (Fig. 3c). The total abundances were at least 2 orders of magnitude higher in animal-associated mucosa than in the free-living communities of surface marine environments (*t* test *P* = 7.02 · 10^−15^ for phage [Fig. 3b], and *P* value = 4.17 · 10^−7^ for bacteria [Fig. 3c]). The most abundant phage genotype (P*_1_*) in marine environments comprised only 0.8% of the total phage community, while in the gut, the dominant phage comprised just over 1% of the community (Fig. 3d). In the bacterial community, this pattern was inverted, with the dominant bacterial species (B*_1_*) reaching 19% in marine environments, but only 15% in the gut (Fig. 3e).

### Lysogeny by coinfection in microbial communities.

**FIG 4:**
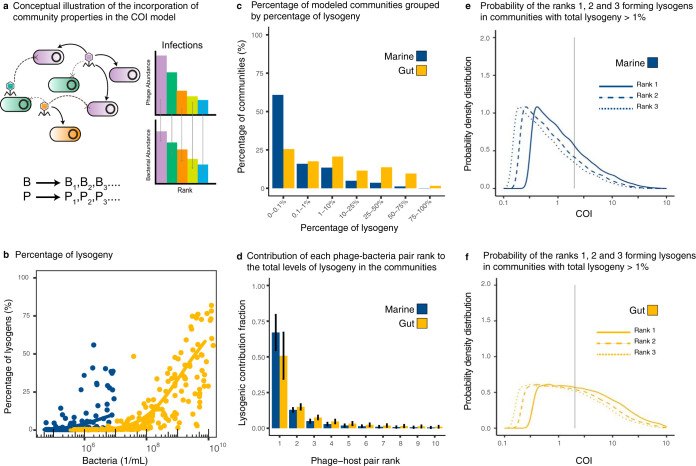
Lysogeny caused by coinfections in communities. (a) Conceptual figure describing the implementation of the biophysical COI model with a stochastic sampling of community parameters. Each phage P*_i_* infected each bacterium B*_i_* according to their ranks in the community. The chances of encounter decreased with phage-host rank as a function of their absolute abundances in the community. (b) Percentage of lysogenic cells in the bacterial community predicted to be formed through phage coinfections as a function of the total bacterial density. The data points are a subsample of 200 stochastic models out of 100,000 sampled models for each ecosystem. The solid lines represent generalized additive models (GAM) fitted to the full data set for marine (blue) and gut (yellow) ecosystems. (c) Percentage of sampled communities displaying different ranges of lysogeny. (d) Average contribution (with error bars corresponding to SD) from the top phage-bacterium pair ranks to the lysogenic pool. (e and f) Probability distributions of average phage coinfections (COI) for the top ranks in communities with lysogeny above 1% for marine and gut ecosystems. The vertical line indicates COI = 2, that is, the average of two phage infections within the commitment time.

The community model assumed a direct phage-host network, where each phage rank infected the same rank in the bacterial community, that is, P*_i_* infected B*_i_* (Fig. 4a). The biophysical COI model quantified the percentage of lysogeny generated by phage coinfections for each pair by stochastically sampling the parameter ranges from the meta-analysis of marine and gut ecosystems. The percentage of lysogeny caused by coinfections increased with total bacterial density (Fig. 4b and Fig. S1). Lysogeny was more frequent in the gut, where 25% of the simulated communities displayed at least 25% of bacteria becoming lysogens by coinfection (Fig. 4c, Table 1, and Table S1). The median percentage of lysogeny in these communities was 47.8%. Given the median bacterial abundances (1.7 · 10^9^ cells/ml), duplication times (4.75 h), and volume of the human colon (400 ml [39]), we estimated that a median of 1.8 · 10^12^ lysogens is potentially formed in the human gut every day via coinfection. Among marine communities, 90% displayed 10% or fewer bacteria becoming lysogens by coinfection (Fig. 4c, Table 2, and Table S1).

**TABLE 1:** Summary statistics for gut communities with lysogeny ≥25%^*a*^

Feature	Unit	Min	1st Qu	Median	Mean	3rd Qu	Max
Bacterial concentration	Cells/ml	5.6 · 10^6^	6.8 · 10^8^	1.8 · 10^9^	2.4 · 10^9^	3.9 · 10^9^	7.6 · 10^9^
Phage concentration	Phages/ml	2.0 · 10^8^	8.0 · 10^8^	1.3 · 10^9^	1.9 · 10^9^	2.2 · 10^9^	1.1 · 10^10^
Phage adsorption rate	ml/h	5.9 · 10^−9^	1.3 · 10^−7^	2.6 · 10^−7^	3.7 · 10^–7^	5.6 · 10^–7^	1.2 · 10^–6^
Lysogenic commitment time	h	0.55	0.74	0.95	0.97	1.19	1.45

a Abbreviations: Min, minimum; Qu, Quartile; Max, maximum.

**TABLE 2:** Summary statistics for marine communities with lysogeny ≥10%^*a*^

Feature	Unit	Min	1st Qu	Median	Mean	3rd Qu	Max
Bacterial concentration	Cells/ml	3.8 · 10^4^	7.9 · 10^5^	2.0 · 10^6^	2.5 · 10^6^	3.9 · 10^6^	6.7 · 10^6^
Phage concentration	Phages/ml	6.2 · 10^5^	6.3 · 10^6^	1.3 · 10^7^	1.6 · 10^7^	2.5 · 10^7^	3.9 · 10^7^
Phage adsorption rate	ml/h	6.2 · 10^–9^	7.8 · 10^–8^	1.6 · 10^–7^	1.7 · 10^–7^	2.5 · 10^–7^	3.7 · 10^–7^
Lysogenic commitment time	h	13	183	352	376	558	807

a Abbreviations: Min, minimum; Qu, quartile; Max, maximum.

10.1128/mSystems.00353-20.1 FIG S1 Lysogeny in marine and gut modeled communities. Estimated concentration (a) and percentage (b) of lysogens in the bacterial community as a function of bacterial concentration. Estimated concentration (c) and percentage (d) of lysogens in the bacterial community as a function of phage concentration. In the four panels, the data plotted are a random sample of 200 modeled communities for each marine (blue) and gut (gold) ecosystem. The solid lines correspond to generalized additive models (GAM) for the full sampling (100,000 communities for each ecosystem). Download FIG S1, EPS file, 1.9 MB. Copyright © 2020 Luque and Silveira. 2020 Luque and Silveira This content is distributed under the terms of the Creative Commons Attribution 4.0 International license.

10.1128/mSystems.00353-20.5 TABLE S1 Percentage of communities sampled falling in each range of percentage of lysogeny. Download Table S1, DOCX file, 0.01 MB. Copyright © 2020 Luque and Silveira. 2020 Luque and Silveira This content is distributed under the terms of the Creative Commons Attribution 4.0 International license.

For communities with lysogeny above 1%, the most abundant phage-host pairs contributed an average of 67% ± 12% (standard deviation [SD]) for marine and 51% ± 16% for gut to the total lysogeny (Fig. 4d and Fig. S2). This was significantly higher than the contribution from the second most abundant phage-host pair, which yielded 13% ± 1% for marine and 15% ± 2% for gut. For communities with lysogeny above 1%, the most abundant phage-host rank displayed median COI of 1.00 (one infection on average) for marine (Fig. 4e, Fig. S2, and Table 3) and COI of 2.35 for gut (Fig. 4f, Fig. S2, and Table 4).

**TABLE 3:** Summary statistics for marine communities with lysogeny ≥1%^*a*^

Feature	Unit	Min	1st Qu	Median	Mean	3rd Qu	Max
Bacterial concentration	Cells/ml	3.8 · 10^4^	4.7 · 10^5^	1.5 · 10^6^	2.1 · 10^6^	3.4 · 10^6^	6.7 · 10^6^
Phage concentration	Phages/ml	1.8 · 10^5^	4.0 · 10^6^	9.1 · 10^6^	1.3 · 10^7^	1.9 · 10^7^	3.9 · 10^7^
Phage adsorption rate	ml/h	1.4 · 10^–9^	4.6 · 10^–8^	1.1 · 10^–7^	1.4 · 10^–7^	2.1 · 10^–7^	3.7 · 10^–7^
Lysogenic commitment time	h	11	109	262	311	489	807
Avg (co)infections (COI), rank 1	–	0.31	0.53	1.00	2.63	2.42	85.84
Avg (co)infections (COI), rank 2	–	0.19	0.33	0.62	1.64	1.51	53.43
Avg (co)infections (COI), rank 3	–	0.15	0.25	0.47	1.24	1.14	40.49

a Abbreviations: Min, minimum; Qu, quartile; Max, maximum; Avg, average; −, unitless.

**TABLE 4:** Summary statistics for gut communities with lysogeny ≥1%^*a*^

Feature	Unit	Min	1st Qu	Median	Mean	3rd Qu	Max
Bacterial concentration	Cells/ml	3.8 · 10^5^	9.2 · 10^7^	4.4 · 10^8^	1.3 · 10^9^	1.9 · 10^9^	7.7 · 10^9^
Phage concentration	Phages/ml	3.1 · 10^7^	2.8 · 10^8^	5.3 · 10^8^	1.0 · 10^9^	1.2 · 10^9^	1.1 · 10^10^
Phage adsorption rate	ml/h	5.9 · 10^–8^	1.2 · 10^–7^	2.6 · 10^–7^	3.7 · 10^–7^	5.6 · 10^–7^	1.2 · 10^–6^
Lysogenic commitment time	h	0.55	0.72	0.92	0.95	1.16	1.45
Avg (co)infections (COI), rank 1	–	0.33	0.88	2.35	7.29	7.19	182.07
Avg (co)infections (COI), rank 2	–	0.21	0.55	1.47	4.56	4.50	113.96
Avg (co)infections (COI), rank 3	–	0.16	0.42	1.12	3.47	3.42	86.64

a Abbreviations: Min, minimum; Qu, quartile; Max, maximum; Avg, average; −, unitless.

10.1128/mSystems.00353-20.2 FIG S2 Lysogeny associated with the dominant phage-host members in marine and gut modeled communities. Estimated phage coinfections (a) and percentage of lysogeny (b) from rank 1 in the bacterial community as a function of total bacterial concentration. Estimated phage coinfections (c) and percentage of lysogeny (d) from rank 1 in the bacterial community as a function of total phage concentration. In the four panels, the data plotted are a random sample of 200 modeled communities for each marine (blue) and gut (gold) ecosystem. The solid lines correspond to generalized additive models (GAM) for the full sampling (100,000 communities for each ecosystem). Download FIG S2, EPS file, 1.9 MB. Copyright © 2020 Luque and Silveira. 2020 Luque and Silveira This content is distributed under the terms of the Creative Commons Attribution 4.0 International license.

### Physical parameters contributing to the formation of lysogens in communities.

**FIG 5:**
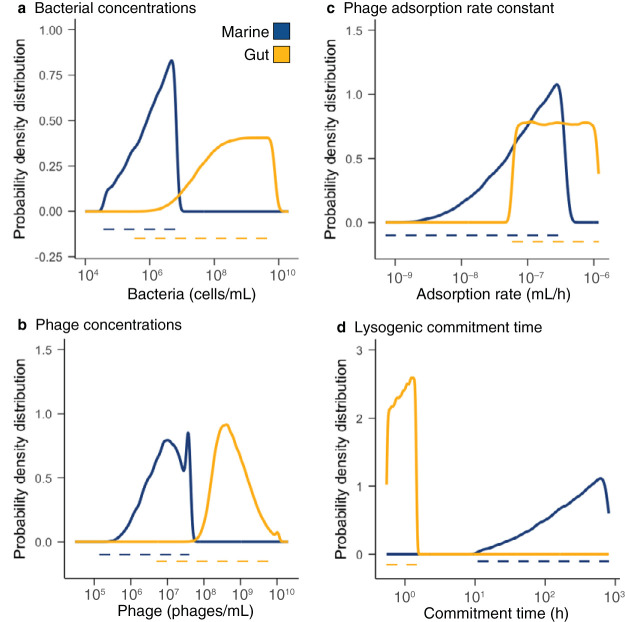
Ranges of parameter values leading to lysogeny caused by coinfection. Probability density distributions (solid lines) for bacterial abundances (a), phage concentrations (b), adsorption rates (c), and lysogenic commitment times (d) in marine (blue) and gut (yellow) communities displaying lysogeny levels above 1%. The dashed lines indicate the range of parameters explored in the model and obtained from the meta-analysis of each ecosystem. The probability densities were calculated with the logarithm in base 10 of the values displayed in the *x* axes.

Communities with at least 1% lysogeny caused by coinfection were analyzed to extract the distribution of physical parameters yielding lysogeny. The distribution of bacterial abundances favoring lysogeny in marine communities was skewed toward high densities with a median of 1.5 · 10^6^ cells/ml (Fig. 5a and Table 3). In gut communities, low bacterial abundances did not contribute to lysogeny, with the first quartile of the probability distribution at 9.2 · 10^7^ cells/ml (Fig. 5a and Table 4). Phage concentrations yielding lysogeny in marine communities were also skewed toward higher densities (median of 9.1 · 10^6^ phages/ml) but more centered than the bacterial density distribution (Fig. 5b and Table 3). In the gut, low phage concentrations did not contribute to lysogeny, displaying a first quartile of the probability distribution at 2.8 · 10^8^ phages/ml (Fig. 5b and Table 4).

Phage adsorption rates in marine communities producing lysogeny were skewed toward high values with a median of 1.1 · 10^−7^ ml/h (Fig. 5c and Table 3). In the gut, instead, the full range of adsorption rates contributed to communities with lysogeny (Fig. 5c and Table 4). The lysogenic commitment time in marine communities producing lysogeny was again skewed toward long time windows, with a median of 262 h (Fig. 5d and Table 3). For the gut, the full range of lysogenic commitment times contributed to producing lysogens, but larger values had a higher likelihood of contribution, displaying a median lysogenic commitment time of 0.92 h (Fig. 5d and Table 4).

## DISCUSSION

The stochastic biophysical COI model introduced here estimated an increase in coinfections in the highly productive mammalian gut microbial communities (Fig. 4). This higher frequency of coinfections predicts considerable levels of lysogeny in animal mucosa microbiomes, as observed empirically (9, 11, 12, 14, 16, 17), and supports the Piggyback-the-Winner framework (15, 16). In the murine gut, empirical genomic data show that 65.8% of the bacterial genomes are lysogens and that 83.2% of the prophages observed in these lysogens are active (40). Together, these lysogens reach relative abundances of 53.6% to 78.6% of the bacterial community. This frequency of lysogeny is consistent with the output of the coinfection model, which predicts lysogeny levels between 40% and 70% for abundances above 10^9^ cells/ml (Fig. 4b, solid line). The model also predicted that 41.89% of the gut communities displaying lysogeny above 1% had virus-to-microbe ratios (VMR) lower than 1 (see Fig. S3 in the supplemental material), indicating that the formation of lysogens through coinfection was compatible with the observation of low virus-to-microbe ratios in high-density animal mucosa (12, 41). This was possible because coinfections were not required to occur simultaneously. Instead, they occurred within the lysogenic commitment time, which was assumed to be proportional to the duplication time of bacteria *in vivo* (42–44).

10.1128/mSystems.00353-20.3 FIG S3 Relationship between percent lysogeny and virus-to-microbe ratios (VMR). The VMRs were calculated from the viral and microbial abundances sampled stochastically for each marine (a) and gut (b) community yielding lysogeny above 1% in the COI model. The vertical line indicates VMR = 1. Download FIG S3, EPS file, 1.5 MB. Copyright © 2020 Luque and Silveira. 2020 Luque and Silveira This content is distributed under the terms of the Creative Commons Attribution 4.0 International license.

The lysogenic commitment time played a paramount role in the findings of the model because the duplication time of gut bacteria *in vivo* is significantly slower than the duplication time of gut bacterial isolates in pure cultures (42–44). For laboratory E. coli duplication times, the model predicted an average number of coinfections almost an order of magnitude lower than that *in vivo* (Fig. 1d). This result aligned with the low number of coinfections observed even at high MOIs in a recent stochastic model that used parameters similar to lambda and E. coli (38). The influence of the lysogenic commitment time was even more pronounced in marine communities (Fig. 5d). Lysogeny levels above 10% were favored for communities that displayed long lysogenic commitment times, which compensated for the lower adsorption rates and phage and bacterial abundances (Fig. 3 and 5).

The relationship between the lysogenic commitment time (τ) and duplication time in the biophysical COI model was based on lambda-E. coli single-cell experiments, which identified the relationship τ ∼20% of the duplication time (25, 45). This pattern arises from the voting phenomenon, where each coinfecting phage genome independently votes for lytic or lysogenic commitment (46). In cells that undergo lysogeny, phages cooperate for host cell resources (25). Late phage genome arrivals contribute less to the cell-level decision, and the time window for the contribution of the second phage is proportional to the transcription level of phage repressors, which vary with the host growth rate (45). Future studies addressing the relationship between the lysogenic commitment time and host duplication time in other phages would refine the model and further test its validity in different ecosystems.

Only 10% of the simulated marine communities displayed levels of lysogeny above 10%. These communities were characterized by high phage and bacterial concentrations, displaying medians of 1.3 · 10^7^ phages/ml and 2.0 · 10^6^ cells/ml (Fig. 5a and b; Table 2). A direct comparison between empirical data and our model outputs was not possible due to the lack of estimates of percent lysogenic bacterial cells in marine environments using genomic data, which remains a bioinformatics challenge. Previous studies assessed the percentage of lysogeny in marine samples using mitomycin C induction, but this method has been proved inaccurate, and the density relationships derived from it are unclear (47). Alternatively, we pursued an indirect approach to compare the results of the model with empirical data by analyzing indicators of lysogeny that were available for both marine and gut ecosystems. In the free viral particle metagenomes, only 5% to 20% of the identified viral contigs are predicted to be temperate in marine samples (7), 3 to 15 times less than that observed in the gut, which ranges from 53% to 72% (40). When comparing genomes from isolates, marine bacteria encode 5 ± 2 (mean ± SD) prophages per genome, three times less than human gut bacteria, which encode 14 ± 5 prophages per genome (17). In the model, marine communities displayed, on average, 5 to 10 times less lysogeny than gut communities (solid lines in Fig. 4b). This ratio is on the same order of magnitude as the change in the two empirical indicators for lysogeny (temperate phage particles and prophage abundances) in marine and gut communities.

The stochastic community simulations assumed that the most dominant phages infected the most dominant bacteria (10, 11, 48–51). This empirically-based assumption allowed us to bridge the environmental data on viral and bacterial abundances with the species distributions from genomic data (Fig. 3). In the model, the most abundant phage-host pairs dominated the formation of lysogens (Fig. 4d and Fig. S2). If temperate phages do not occupy the first rank, lysogeny via coinfection will decrease by about 50% in marine and 70% in gut communities, unless there is a cross-infection network. Metagenomic data support that temperate phages and their hosts are likely to occupy high ranks in the community. For example, pelagiphages infecting SAR11, the most abundant marine bacterium, account for 35% of the phage particles in the virioplankton, representing the most abundant phages in the oceans, and 11 out of 16 isolated pelagiphages are temperate (50, 52, 53). If these closely related phages cooperate during the lysogenic decision, their summed abundances would be much higher than the abundance of the highest rank in our model, 0.8% for marine communities. One challenge in building accurate phage-bacterial infection networks to test these comparisons is that the hosts of the majority of phages identified from metagenomic analyses are unknown (8, 54, 55). The reconstruction of accurate infection networks will, in the future, improve the model’s predictive power on the contribution of coinfections to lysogeny (56, 57).

The average percentage of lysogens formed by coinfection decreased by almost 2 orders of magnitude as total bacterial concentrations dropped from 10^6^ to 10^5^ cells/ml (Fig. S1b). This contrasts with viral metagenomic studies from deep oceans with microbial abundances ranging from 10^4^ to 10^5^ cells/ml showing an increase in lysogeny compared to more productive surface waters (7, 20). In these environments, lysogeny has been proposed to serve as a low-density refugium for temperate phages in conditions of poor host growth and scarce resources for viral particle production (58–61). In the biophysical COI results, lysogeny in these low-cell-density communities occurred at very long commitment times (Fig. S4), which is likely the case in the natural environment. The model did not incorporate assumptions relating bacterial densities and commitment times. Adding this relationship would increase the percentage of lysogeny predicted in deep oligotrophic waters. Additional mechanisms could also contribute to the increase of lysogeny at these low concentrations, such as the favored phage integration in starved cells as observed in lambda due to the reduced degradation of the lytic repressor (62).

10.1128/mSystems.00353-20.4 FIG S4 Relationship between commitment time (τ) and bacterial densities. Commitment time and bacterial densities sampled by the stochastic model of each marine (a) and gut (b) community yielding lysogeny above 1%. The color gradient indicates the percentage of lysogeny obtained from phage coinfections. Download FIG S4, JPG file, 0.1 MB. Copyright © 2020 Luque and Silveira. 2020 Luque and Silveira This content is distributed under the terms of the Creative Commons Attribution 4.0 International license.

The COI model assumed that two phage infections occurring within the commitment time were necessary for lysogeny. This assumption was based on the observation that most temperate phages seem to encode a repressor system that is functionally similar to lambda’s cro/cI (33, 34). This includes phages in marine environments, such as temperate phages infecting SAR11, suggesting that lessons learned from lambda can be extended to the marine environment (7, 52, 53). The model introduced here, however, does not capture lysogeny from a smaller fraction of temperate phages, such as P1 and P4-like, that do not respond to coinfections (63, 64). Further work will be necessary to assess alternative mechanisms for the control of lysogeny and refine the model predictions.

The lysogenic commitment time provides plasticity for phage adaptation to different ecosystems, which may be the reason why the response to coinfection has been selected in disparate environments. Phage densities are higher in the gut where bacterial replication times (and commitment times) are shorter than in marine environments. Above environment-specific density thresholds, communities would be driven to extinction by lysis unless immunity mechanisms emerge (65). Because of its superinfection immunity, lysogeny could be selected through the plastic coinfection response that depends on growth rates. Other density-dependent mechanisms, such as quorum sensing, may also contribute to maintaining population stability when phage densities are relatively high (66, 67). Coinfections might also act together with other molecular defenses, such as bacterial restriction-modification systems, which delay infection onset until bacteria reach densities that favor lysogeny via coinfection (68). Our model did not incorporate mechanisms that would lead to stability over long-term evolutionary dynamics (69). Further work will be necessary to assess these stability mechanisms.

### Conclusion.

The stochastic biophysical COI model proposed here identified the ranges of physical parameters that drive phage coinfections in complex microbial communities. The model predicted a high frequency of lysogeny caused by phage coinfections in the mammalian gut. This finding was a consequence of high phage and bacterial densities and high phage adsorption rates in comparison with marine communities. Longer lysogenic commitment times *in vivo*, compared to laboratory isolates, also contributed to high lysogeny in the gut. The simulated marine communities showed a lower frequency of lysogeny by coinfections. Those communities that displayed a high fraction of lysogeny were characterized by long lysogenic commitment times. Our findings bridge the main molecular mechanism causing lysogeny in laboratory systems with metagenomic observations of lysogeny in complex microbial communities.

## MATERIALS AND METHODS

### Phage coinfection model.

The average number of phage (co)infections (COI) was derived from physical properties of phage and bacteria (Fig. 1a). The rate of phage infections on a single bacterium can be estimated by solving the Smoluchowski coagulation equation (37). In a well-mixed community, this rate is the product of the phage concentration (P*_i_*) and the phage adsorption rate (α), which depends on the mobilities and sizes of both the phage particle and the bacterium. The adsorption rate constant (α) expresses how fast a single phage adsorbs to a single bacterium given a volume (37), and its units are expressed here in ml/h. The subindex *i* specifies a single phage-bacterium pair in the community.

The number of (co)infections (COI) was defined as the number of phages infecting a cell within a given time window. This number was the product of the infection rate and the time window. In the case of lysogeny, this time corresponds to the lysogenic commitment time (τ), when the second phage can still interfere with the decision (lysis or lysogeny) of the first infecting phage (25). This led to the average phage coinfection equation (Fig. 1a)

(1) $$\text{COI}={\text{P}}_{i}\cdot \text{α}\cdot \text{τ}$$

Therefore, COI = 1 means one infection per cell on average within the window time, and COI = 2 means two phage infections. The average probability of coinfections was calculated assuming that each infection was independent and that, in a given environmental community, the changes in phage concentration (P*_i_*) and bacterial concentrations (B*_i_*) were small (within 20%) during the lysogenic commitment times (τ). This assumption is consistent with the typical changes of abundances in the environment (58, 70), but it does not apply during rapid changes in abundances observed under laboratory conditions (38). In the community model described below, the variance in COI due to the variance in (P*_i_*) and (B*_i_*) in a given community is negligible compared to the variance in COI resulting from the stochastic sampling across the ranges of microbial traits (see meta-analysis and stochastic sampling sections below).

The average number of coinfections was also expressed as a function of the phage-to-bacterium ratio, COI = α · τ · B*_i_* · (P*_i_*/B*_i_*), as a proxy for comparison with lambda-to-E. coli ratio in MOI experiments. Numerically, the phage-to-bacterium ratio (P*_i_*/B*_i_*) was explored for the range 0.01 to 100. The median values extracted for the lambda adsorption rate (α_0_ = 5.6 · 10^−7^ ml/h), lysogenic commitment time (τ_0_ = 0.1 h), and bacterial concentration (B_0_ = 5 · 10^8^ cells/ml) were used as reference values (see section on meta-analysis for lambda parameters). Two parameters were fixed at these reference values, and the third was explored over a range of values based on the meta-analysis of microbial communities (see details below). These ranges were 10^5^ to 10^10^ cells/ml for bacterial concentrations, 10^−11^ to 10^−6^ ml/h for the phage adsorption rates, and 10^−3^ to 10^2^ h for the lysogenic commitment times.

### Percentage of lysogeny for the coinfection model.

A lysogen was formed when a cell was infected within the lysogenic commitment time by two or more phages from the same phage-host pair. This was based on the effect of cooperative infection by phages on the production of lysogens (24, 25, 46). Thus, the probability of lysogenization, *p*_lys_, was determined by *p*_lys_ = 1 − *p*(0) − *p*(1), where *p*(*k*) was the probability of having *k* infections within the commitment time. The probability of *k* infections with average (co)infection COI, equation 1, was given by a Poisson distribution *p*(*k*) = COI*^k^*e^−COI^/*k*!. The probability of forming a lysogen via coinfection was

(2) $$p_{\text{lys}}=1-{\text{e}}^{-\text{COI}}-{\text{COI e}}^{-\text{COI}}$$

The model assumed that a higher probability of lysogenization resulted in a higher prevalence of lysogeny. This assumption was supported by experimental data (68, 69).

The probability of lysogenization was compared with the percentage of lysogeny obtained from lambda and E. coli MOI experiments (28, 29). The empirical data for the percentage of lysogeny and MOI were fitted using the nonlinear least-squares method for Hill-Langmuir cooperation models, *f*(*x*) = *ax*/(*b* + *x^n^*), with cooperation orders *n* = 1, *n* = 2, and *n* = 3.

### Meta-analysis of marine and gut microbiomes.

**(i) Adsorption rates.** The adsorption rates were obtained from 71 prior experiments using 19 phage-host pairs from marine and gut microbiomes (see Data Set S1 in the supplemental material). The data consisted of values for tailed phages infecting E. coli (37, 71), *Synechococcus* sp. (72–74), *Prochlorococcus* sp. (75–77), *Vibrio* sp. (78–81), *Roseobacter* sp. (82), and *Pseudoalteromonas* sp. (48). A *t* test (double-tailed) compared the marine and gut values.

10.1128/mSystems.00353-20.6 DATA SET S1 Adsorption rates obtained from 71 prior experiments using 19 phage-host pairs from marine and gut microbiomes. Download Data Set S1, CSV file, 0.003 MB. Copyright © 2020 Luque and Silveira. 2020 Luque and Silveira This content is distributed under the terms of the Creative Commons Attribution 4.0 International license.

**(ii) Lysogenic commitment times.** The lysogenic commitment time (τ) was assumed to be 20% of the bacterial duplication time (25, 45). The ranges of bacterial duplication times were obtained from *in situ* data sets for marine ecosystems (83) and from *in vivo* data sets for mammalian gut ecosystems (42–44) (Data Set S2).

10.1128/mSystems.00353-20.7 DATA SET S2 Ranges of bacterial duplication times and estimated commitment times obtained from *in situ* data sets for marine ecosystems and from *in vivo* data sets for mammalian gut ecosystems. Download Data Set S2, CSV file, 0.01 MB. Copyright © 2020 Luque and Silveira. 2020 Luque and Silveira This content is distributed under the terms of the Creative Commons Attribution 4.0 International license.

**(iii) VLPs and cell abundances.** Direct counts of virus-like particles (VLPs) and microbial cells were obtained for marine surface waters (15, 84) and animal-associated microbiomes (41, 85–88) (Data Set S3). A *t* test (double-tailed) compared the concentrations of VLPs and cells between marine and animal ecosystems.

10.1128/mSystems.00353-20.8 DATA SET S3 Direct counts of virus-like particles (VLPs) and microbial cells obtained for marine surface waters and animal-associated microbiomes. Download Data Set S3, CSV file, 0.1 MB. Copyright © 2020 Luque and Silveira. 2020 Luque and Silveira This content is distributed under the terms of the Creative Commons Attribution 4.0 International license.

**(iv) Phage and bacterial diversity.** The rank-abundance curves of phage genotypes were constructed from the median slope and intercept of power-law functions fitted to 192 marine viromes and 1,158 human-associated viromes (89) (Data Set S4). Phage genotypes were defined as unique viral contigs at 98% sequence identity. The rank-abundance curves of bacterial species in marine communities were obtained from operational taxonomic unit (OTU) tables constructed by clustering universal, protein-coding, single-copy phylogenetic marker genes into metagenomic OTUs (which can be interpreted as species-level clusters) from the Tara Oceans data set (Data Set S5) (90, 91). For animal-associated bacterial microbiomes, rank-abundance curves were constructed using OTU tables obtained by mapping metagenomic reads from 11,850 human gut metagenomes to 92,143 metagenome-assembled genomes (92). Consensus rank-abundance curves were obtained by averaging the frequency of bacteria in the same rank across the metagenomes within each ecosystem.

10.1128/mSystems.00353-20.9 DATA SET S4 Rank-abundance curves of phage genotypes constructed from the median slope and intercept of power-law functions fitted to 192 marine viromes and 1,158 human-associated viromes. Download Data Set S4, CSV file, 0.02 MB. Copyright © 2020 Luque and Silveira. 2020 Luque and Silveira This content is distributed under the terms of the Creative Commons Attribution 4.0 International license.

10.1128/mSystems.00353-20.10 DATA SET S5 Rank-abundance curves of bacterial species in marine communities from operational taxonomic unit (OTU) tables constructed by clustering universal, protein-coding, single-copy phylogenetic marker genes into metagenomic OTUs (which can be interpreted as species-level clusters) from the Tara Oceans data set. For animal-associated bacterial microbiomes, rank-abundance curves were constructed using OTU tables obtained by mapping metagenomic reads from 11,850 human gut metagenomes to 92,143 metagenome-assembled genomes. Consensus rank-abundance curves were obtained by averaging the frequency of bacteria in the same rank across the metagenomes within each ecosystem. Download Data Set S5, CSV file, 0.002 MB. Copyright © 2020 Luque and Silveira. 2020 Luque and Silveira This content is distributed under the terms of the Creative Commons Attribution 4.0 International license.

### Quantification of lysogeny through phage coinfection in communities.

The biophysical COI model, equations 1 and 2, was applied to predict the probability of lysogenization in marine and gut ecosystems as a result of coinfection. The model generated stochastic communities that sampled empirical phage and bacterial concentrations, relative abundance of the top 100 members of the community, phage adsorption rates, and lysogenic commitment times obtained from the meta-analysis of marine and gut ecosystems described above.

**(i) Stochastic sampling.** The model generated 100,000 stochastic communities for both marine and gut ecosystems using Latin hypercube sampling (LHS). For each ecosystem, the ranges of bacterial concentrations, adsorption rates, and lysogenic commitment times were each divided into equal intervals in logarithmic scale (base 10), generating 100,000 points per coordinate. These coordinates defined the hypercube. One hundred thousand random values were sampled from the hypercube without repeating any coordinate value, that is, all coordinate values were sampled, following the standard LHS implementation (17, 93, 94).

**(ii) Parameter ranges.** The ranges of bacterial concentrations used were 3.78 · 10^4^ to 6.75 · 10^6^ bacteria/ml for marine communities and 3.45 · 10^5^ to 7.60 · 10^9^ bacteria/ml for gut. The ranges of phage concentrations used were 1.45 · 10^5^ to 3.80 · 10^7^ phages/ml for marine and 5.09 · 10^6^ to 1.05 · 10^10^ phages/ml for gut. The ranges of phage adsorption rate constants used were 7.2 · 10^−10^ to 3.7 · 10^−7^ ml/h for marine and 5.9 · 10^−8^ to 1.2 · 10^−6^ ml/h for gut. The ranges of lysogenic commitment times used were 11 h to 808 h for marine and 2.74 h to 7.27 h for gut. All parameter ranges were obtained in the meta-analysis described above.

**(iii) Assumed relationships.** Based on environmental data of microbial communities, the total phage concentration (P) was modeled following a power function relationship with the total bacterial concentration (B) (15, 17, 39, 95): P(B) = *a* (B/B*_u_*)*^b^*. The bacterial concentration was given in units of B*_u_* = bacteria/ml. The prefactor *a* and exponent *b* were obtained by fitting the power function to the viral and microbial counts obtained in the marine and gut meta-analyses. A linear regression fit was applied using the least-squares method to the log-log data in base 10. The parameters obtained were *a* = 10^2.50^ phage/ml and *b* = 0.712 for marine and *a* = 10^5.35^ phage/ml and *b* = 0.388 for gut. To reproduce the noise observed in empirical communities, the value log_10_ P(B) was weighted by a normal distribution, N(mean, SD), with mean 1 and standard deviation 0.05 in logarithmic space (base 10), that is, log_10_P = N(1,0.05) · log_10_P(B). The final value of the phage concentration was constrained within the empirical phage abundance range, that is, P_min_ ≤ P ≤ P_max_. The community model also assumed that the most dominant phages infected the most dominant bacteria (10, 11, 48–51). This led to a phage-host network where the phage of rank *i* infected the bacteria with the same rank *i*.

### Data availability.

The codes for the model are available in the GitHub repository at https://github.com/luque82/Luque_and_Silveira_2020.git.
