# Supplementary material for: Quantification of Lysogeny Caused by Phage Coinfections in Microbial Communities from Biophysical Principles
Source: mSystems. 2020 Sep 15;5(5):e00353-20. doi: 10.1128/mSystems.00353-20 (PMC7498681; doi:10.1128/mSystems.00353-20)
Supplement: TABLE S1 [file mSystems.00353-20-st001.docx]

**Table S.1.** Percentage of communities sampled falling in each range of percentage of lysogeny.

| **Lysogeny percentage range** | **Marine communities (%)** | **Gut**  **communities (%)** |
| --- | --- | --- |
| 0–0.1 % | 60.8 | 25.7 |
| 0.1–1 % | 16.0 | 17.5 |
| 1–10 % | 13.5 | 20.4 |
| 10–25 % | 4.8 | 11.5 |
| 25–50 % | 3.7 | 13.4 |
| 50–75 % | 1.1 | 9.8 |
| 75–100 % | 0.1 | 1.6 |
