# Supplementary figures and images for: Quantification of Lysogeny Caused by Phage Coinfections in Microbial Communities from Biophysical Principles
Source: mSystems. 2020 Sep 15;5(5):e00353-20. doi: 10.1128/mSystems.00353-20 (PMC7498681; doi:10.1128/mSystems.00353-20)

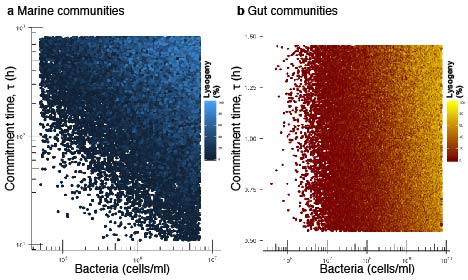

Supplement: FIG S4 [file mSystems.00353-20-sf004.jpg]
